# Supplementary figures and images for: Genetic architecture study of rheumatoid arthritis and juvenile idiopathic arthritis
Source: PeerJ. 2020 Jan 15;8:e8234. doi: 10.7717/peerj.8234 (PMC6969553; doi:10.7717/peerj.8234)

## Legends

### 1. Gene score

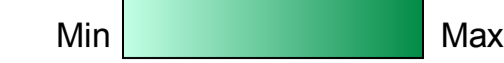

## 2. Interaction score

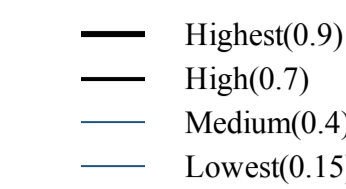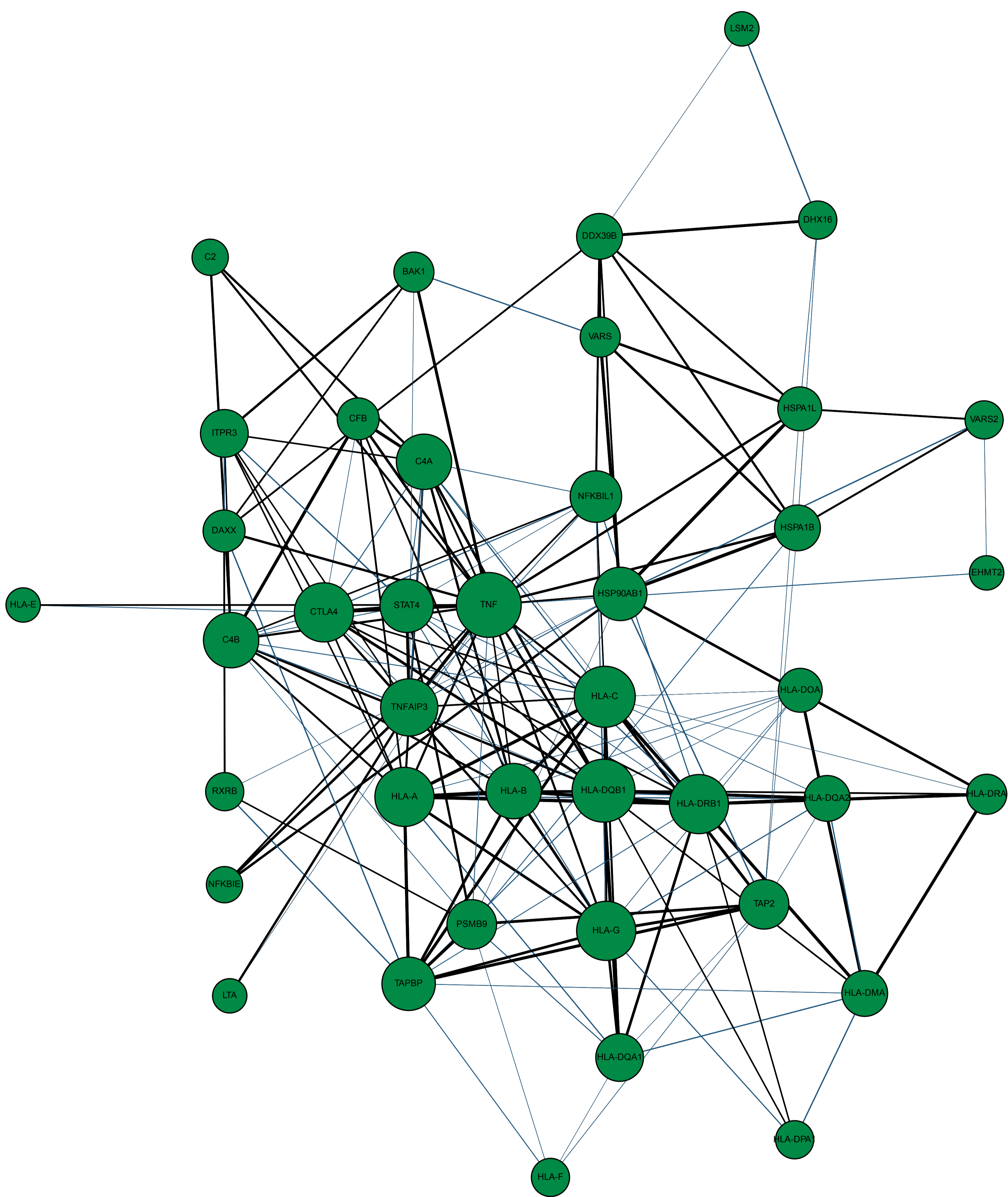

Supplement: Figure S1 — The PPI network was constructed among proteins encoded by the significant RA-associated genes including those in the HLA region. The nodes in the figure represent the proteins and the connections between nodes indicate protein-protein interactions. The size of each node suggests the degrees of the connection between the node and the others. [file peerj-08-8234-s008.pdf]

**Legends**

**1.Gene score**

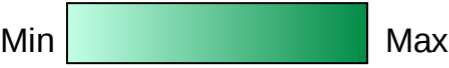

**2.Interaction score**

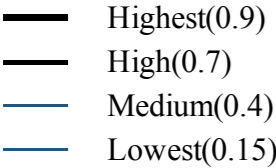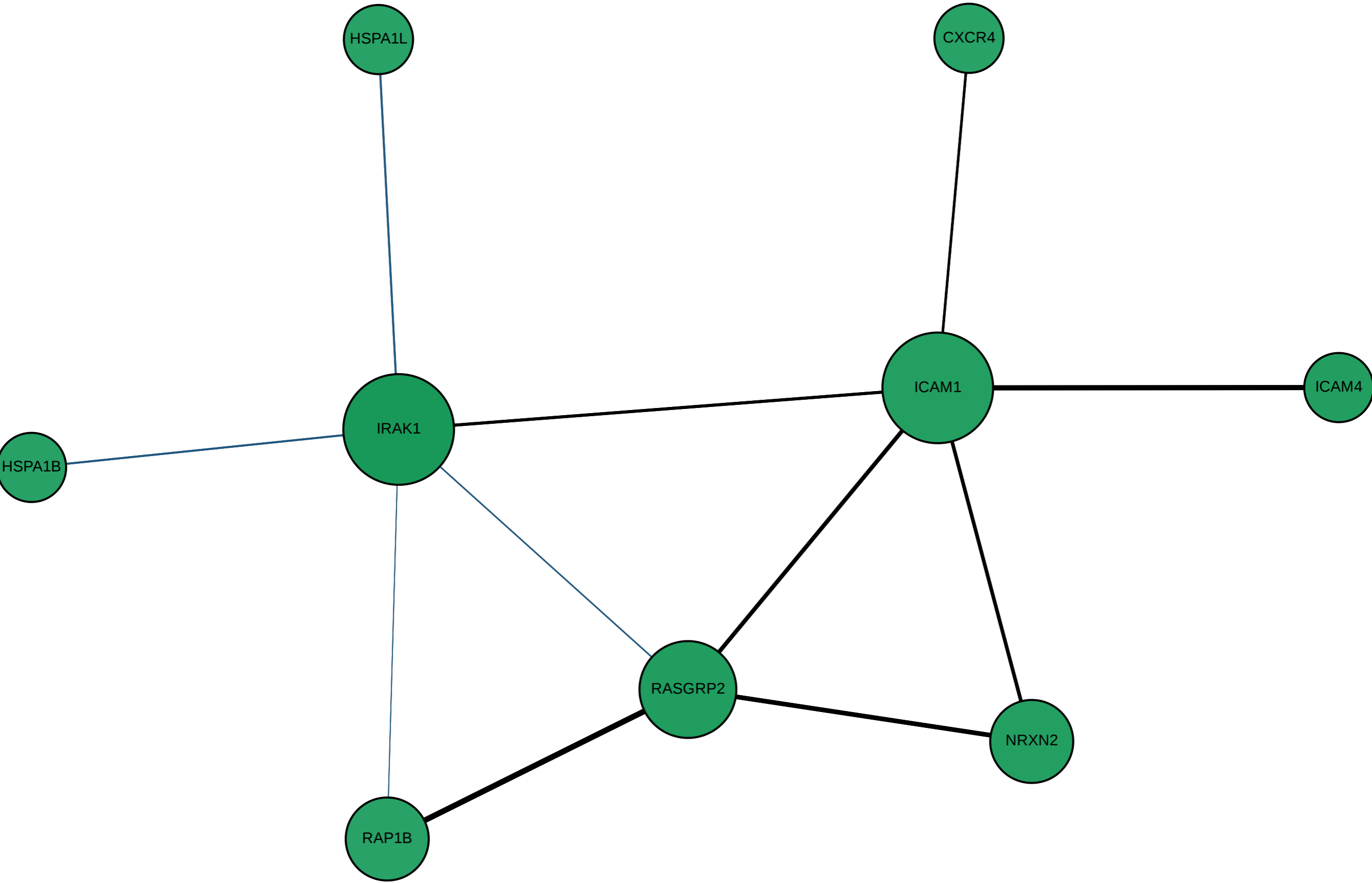

Supplement: Figure S2 — The PPI network was constructed among proteins encoded by the significant JIA-associated genes including those in the HLA region. The nodes in the figure represent the proteins and the connections between nodes indicate protein-protein interactions. The size of each node suggests the degrees of the connection between the node and the others. [file peerj-08-8234-s009.pdf]
